# Methicillin-resistant *Staphylococcus aureus* and coagulase-negative *Staphylococcus* produce antimicrobial substances against members of the skin microbiota in children with atopic dermatitis

**DOI:** 10.1093/femsec/fiae070

**Published:** 2024-05-28

**Authors:** Lorrayne Cardoso Guimarães, Gizele Duarte Garcia, Fernanda Sampaio Cavalcante, Graciela Maria Dias, Felipe Miceli de Farias, Simone Saintive, Eliane de Dios Abad, Dennis de Carvalho Ferreira, Kátia Regina Netto dos Santos

**Affiliations:** Departamento de Microbiologia Médica, Instituto de Microbiologia Paulo de Góes, Universidade Federal do Rio de Janeiro, Rio de Janeiro, Brazil; Departamento de Clínica Médica, Instituto de Ciências Médicas, Universidade Federal do Rio de Janeiro, Campus Macaé, Macaé, Rio de Janeiro, Brazil; Departamento de Clínica Médica, Instituto de Ciências Médicas, Universidade Federal do Rio de Janeiro, Campus Macaé, Macaé, Rio de Janeiro, Brazil; Laboratório de Física Biológica, Instituto de Biofísica Carlos Chagas Filho, Universidade Federal do Rio de Janeiro, Rio de Janeiro, Brazil; APC Microbiome Ireland, University College Cork, Cork, Ireland; Ambulatório de Dermatologia Pediátrica, Instituto de Puericultura e Pediatria Martagão Gesteira, Universidade Federal do Rio de Janeiro, Rio de Janeiro, Brazil; Ambulatório de Dermatologia Pediátrica, Instituto de Puericultura e Pediatria Martagão Gesteira, Universidade Federal do Rio de Janeiro, Rio de Janeiro, Brazil; Faculdade de Odontologia, Universidade Estácio de Sá, Rio de Janeiro, Brazil; Faculdade de Odontologia, Universidade Veiga de Almeida, Rio de Janeiro, Brazil; Faculdade de Enfermagem, Departamento de Fundamentos de Enfermagem, Universidade do Estado do Rio de Janeiro, Rio de Janeiro, Brazil; Departamento de Microbiologia Médica, Instituto de Microbiologia Paulo de Góes, Universidade Federal do Rio de Janeiro, Rio de Janeiro, Brazil

**Keywords:** antimicrobial substance, atopic dermatitis, bacteriocin, coagulase-negative *Staphylococcus*, skin microbiota, *Staphylococcus aureus*

## Abstract

Coagulase-negative *Staphylococcus* (CoNS) species inhibiting *Staphylococcus aureus* has been described in the skin of atopic dermatitis (AD) patients. This study evaluated whether *Staphylococcus* spp. from the skin and nares of AD and non-AD children produced antimicrobial substances (AMS). AMS production was screened by an overlay method and tested against NaOH, proteases and 30 indicator strains. Clonality was assessed by pulsed-field gel electrophoresis. Proteinaceous AMS-producers were investigated for autoimmunity by the overlay method and presence of bacteriocin genes by polymerase chain reaction. Two AMS-producers had their genome screened for AMS genes. A methicillin-resistant *S. aureus* (MRSA) produced proteinaceous AMS that inhibited 51.7% of the staphylococcal indicator strains, and it was active against 60% of the colonies selected from the AD child where it was isolated. On the other hand, 57 (8.8%) CoNS from the nares and skin of AD and non-AD children, most of them *S. epidermidis* (45.6%), reduced the growth of *S. aureus* and other CoNS species. Bacteriocin-related genes were detected in the genomes of AMS-producers. AMS production by CoNS inhibited *S. aureus* and other skin microbiota species from children with AD. Furthermore, an MRSA colonizing a child with AD produced AMS, reinforcing its contribution to dysbiosis and disease severity.

## Teaser

Methicillin resistant Staphylococcus isolated from skin of atopic dermatitis children produces antimicrobial substances which could contribute to the dysbiosis and the disease severity.

## Introduction

Atopic dermatitis (AD) is a chronic, inflammatory skin disease and one of the main dermatological conditions that can occur in the pediatric population around the world. Its pathogenesis is not fully understood, but besides genetic features and impaired immunological system and cutaneous barrier, altered skin microbiota is associated with this condition (Weidinger et al. 2018). Even though it is agreed that *S. aureus* is a major pathogen in AD (Totté et al. 2016), few studies evaluate the role of coagulase-negative *Staphylococcus* (CoNS) in this disease (Cau et al. 2020).

Regarding AD skin microbiota, it is reported that 60% of the AD patients are colonized by *S. aureus* on their nares and 70% on skin lesions (Totté et al. 2016). It is of note that some strains present virulence factors that may increase inflammation in the AD skin (Cavalcante et al. 2021). Furthermore, during crises, in which the patient presents exacerbation of symptoms, such as skin lesions and itching, a reduction of the bacterial diversity is observed, along with an increase in *S. aureus* counts on the skin lesions (Byrd et al. 2017). The high abundance of *S. aureus* in the skin of AD patients has been associated with greater disease severity (Byrd et al. 2017). In addition, frequent recolonization by *S. aureus* between nares and skin has been observed, which can contribute to the severity of AD (Chiu et al. 2009).

In some individuals, during crises, the increase in the abundance of *S. aureus* in the lesions is accompanied by an increase of *S. epidermidis* abundance (Byrd et al. 2017). However, even though strains of this species have been reported inhibiting *S. aureus* growth in AD patients (Nakatsuji et al. 2017), it has been found that *S. epidermidis* from the AD skin produces higher amounts of a protease associated with the skin damage (Cau et al. 2020). Recently, our group also observed a high prevalence of methicillin-resistant CoNS isolates in the skin and nares of AD children, which could contribute to antimicrobial resistance dissemination (Guimarães et al. 2022). Furthermore, a lower prevalence of skin and nasal colonization by *S. hominis*, a species commonly described as beneficial, was described (Guimarães et al. 2022). In fact, the use of a lotion containing a strain of *S. hominis* able to inhibit *S. aureus* demonstrated promising results in a clinical trial (Nakatsuji et al. 2021b). Therefore, the exact role of CoNS in AD is yet not fully understood.

Studies report the presence of *Staphylococcus* spp. strains expressing molecules that control microbial growth and virulence (Iwase et al. 2010, Zipperer et al. 2016, Williams et al. 2019, O'Sullivan et al. 2019a,b). The production of antimicrobial substances (AMS) by members of this genus, such as bacteriocins, ribosomally synthesized antimicrobial peptides active against related bacterial species, could promote healthy skin and nasal microbiota of humans through competition with pathogens (Janek et al. 2016, Zipperer et al. 2016, Liu et al. 2020). In fact, a study found a lower prevalence of CoNS producing AMS that inhibit *S. aureus* colonizing the skin of adults with AD, which could be correlated to the high counts of *S. aureus* in the skin of this population (Nakatsuji et al. 2017).

Since interaction between *Staphylococcus* species in AD is scarcely assessed, this study aimed to evaluate AMS production by *Staphylococcus* species colonizing the skin and nares of Brazilian AD and non-AD children. One of the main novelties of this research was the evaluation of the AMS activity against members of the patient skin microbiota. To the best of our knowledge this is the first study to assess production of AMS by *S. aureus* isolated from AD children, which could add another explanation about the high abundance of this pathogen in the altered skin of AD patients.

## Materials and methods

### Ethics approval, study design, and sample acquisition

The study population included 42 children aged 2 to 10 years: 30 with AD and 12 siblings of AD children without the disease. The design of this study, sample collection, bacterial isolation and identification are described in a recent study of our group as the present article is a continuation of that study (Guimarães et al. 2022). The study followed ethical guidelines and good laboratory practice. A formal consent/assent was obtained from all the legal guardians/patients enrolled in the study (approved by the ethics committee of the Instituto de Puericultura e Pediatria Martagão Gesteira Nº3493662). Up to five mannitol fermenting and ten non-fermenting colonies were selected from each skin specimen, and one colony of each morphology from the nares of each child. Bacterial colonies were identified by MALDI-TOF MS and PCR methods (Guimarães et al. 2022).

### Screening for AMS production

AMS production was evaluated as described by Giambiagi-Marval et al. (1990), with minor modifications. Three microliters of the CoNS isolates and 2 µl of the *S. aureus* isolates previously grown in brain-heart infusion, BHI (Becton, Dickinson and Company; Sparks, MD, USA), were inoculated in spots in BHI agar (Becton, Dickinson and Company; Sparks, MD, USA) and incubated at 35ºC for 24 h. These isolates were killed by exposing them to chloroform for 30 min. Following this procedure, the plates were left open for 30 min in order to evaporate the residual chloroform. Then, 1:100 of the indicator strain previously grown in BHI and adjusted to the 0.5 or 1.0 McFarland scales was inoculated in BHI containing 0.75% agar, and this suspension was immediately overlaid in the plate, which was incubated at 35ºC for 24 h. Any zone of reduction of the indicator strain around the spots was considered as possible AMS production. The indicator strains were: *Staphylococcus aureus* ATCC29213 (adjusted to the 0.5 McFarland scale), when CoNS isolates were screened; and *Staphylococcus epidermidis* ATCC12228 (adjusted to the 1.0 McFarland scale), when *S. aureus* isolates were evaluated.

### Characterization of the AMS

The proteinaceous or acidic nature of the AMS were evaluated according to Giambiagi-Marval et al. (1990). In this regard, 40 µl of each of the following reagents (Sigma Chemical Company; Saint Louis, MO, USA) were added around the spots and incubated at 35ºC for 4 h prior to the indicator strain overlaid: 0.2 M NaOH, 1 mg mL^−1^ proteinase K, 1 mg mL^−1^ pronase, and 1 mg mL^−1^ trypsin. Isolates that did not present a decrease in the inhibition zone following the treatment with NaOH were considered as AMS producers, since the growth inhibition of the indicator strain was probably not due to metabolic acid or organic acid production. A decrease in the inhibition zone by at least one protease indicated that the AMS produced was a protein. This assay was performed twice.

### Spectrum of action of the AMS

All the AMS-producers were evaluated for their spectrum of action against 30 indicator strains (Supplementary Table 1) using the same methodology described for AMS screening. The indicator strains included representatives (clinical and reference strains) of the six staphylococcal species more frequently isolated on the skin of the AD and non-AD children (Guimarães et al. 2022) and a strain of *Micrococcus luteus*, a species known for being highly susceptible to staphylococcal bacteriocins (Bastos et al. 2020). The *M. luteus* strain was adjusted to the 1.0 McFarland scale. The *S. aureus* clinical indicator strains were randomly selected among those from the lesional skin of AD children and adjusted to the 0.5 McFarland scale, while CoNS clinical isolates were from both skin sites (with or without lesion) and adjusted to the 1.0 McFarland scale. The test was considered positive when a complete inhibition zone was observed around the spots.

### Clonal evaluation of AMS-producers

AMS-producing colonies of the same species identified in the same child were evaluated for their clonal relatedness through pulsed-field gel electrophoresis (PFGE) as previously described (Vivoni et al. 2006). The whole bacterial DNA was digested with 20 U *Sma*I (New England Biolabs; Ipswich, England) restriction enzyme and the fragments were separated in a CHEF DR III (Bio-Rad; Hercules, CA, USA) apparatus. For *S. hominis* isolates, a digestion with 10 U *Apa*I (New England Biolabs; Ipswich, Inglaterra) restriction enzyme was performed previously to 20 U *Sma*I. The PFGE fingerprints were analyzed with Bio-Numerics 7.6.3 software (Applied Maths, Biomérieux; Sint-Martens-Latem, Belgium). A dendrogram was generated through an unweighted pair group method with arithmetic averages (UPGMA) using the Dice similarity coefficient. Isolates presenting a similarity coefficient ≥ 80% were assigned as the same genotype (Van Belkum et al. 2009).

### Assessment of the interaction of the AMS-producing *S. aureus* strain with colonies selected from the skin and nares of the child 1 with AD

In a first experiment, the AMS-positive colony (23ad, a methicillin-resistant *S. aureus* isolate, MRSA, from child 1) was used as an indicator strain to assess whether the remaining colonies selected from the child 1 with AD produced any AMS against it. In a second experiment, it was evaluated whether the AMS-positive MRSA isolate (23ad) was able to inhibit 10 randomly selected colonies from child 1 (Supplementary Table 2). Both experiments were carried out according to the methodology described for the screening of AMS production. Tests were considered positive when complete inhibition zones were observed around the spots.

### Detection of genes related to *S. epidermidis* bacteriocins

Since bacteriocins are small peptides, PCR was conducted for all proteinaceous AMS-producers to detect four bacteriocin genes of *S. epidermidis* (Ceotto et al. 2009): epicidin 280, epidermin, epilancin K7, and Pep5. The DNA was obtained according to Pitcher et al. (1989). The DNAs used for positive controls were from the isolates: *S. epidermidis* BN280 (epicidin 280) (Heidrich et al. 1998); *S. epidermidis* Tü3298 (epidermin) (Allgaier et al. 1986); *S. epidermidis* K7 (epilancin K7) (van de Kamp et al. 1995); and *S. epidermidis* 5 (Pep5) (Ersfeld-Dressen et al. 1984).

### Auto-immunity assay

Bacteriocin producers tend to present immunity against their own bacteriocins (Bastos et al. 2020). Therefore, we performed an autoimmunity test for 14 randomly selected protein AMS-producing isolates from each isolation site per child (Supplementary Table 3). This test was carried out in duplicated as previously described for screening of AMS production, using the AMS producer as the indicator strain. Isolates were considered immune to the action of its own AMS when a reduction in its own growth was absent. This experiment was performed twice.

### Genome sequencing of two AMS-producing isolates

Genome sequencing was performed for two AMS-producers, MRSA 23ad (selected due to its importance as a pathogen in AD) and *S. epidermidis* 84ad (selected for presenting AMS with a broad spectrum of action). The genomes were submitted to Genbank under the accession numbers SA23-JAUDYF000000000 and SE84-JAUDYG000000000, respectively. DNA was extracted using the DNeasy PowerSoil Pro kit (QIAGEN; Hilden, Germany), following the manufacturer's recommendations. The DNA was quantified using NanoDrop (Thermo Fisher Scientific; Waltham, Massachusetts, USA) and Qubit 4 with the DNA High Sensitivity kit (Thermo Fisher Scientific) and stored at −20ºC until use. Genomic libraries were assembled using the Nextera DNA Flex Library Prep protocol (Illumina; San Diego, CA, USA). Library concentrations were evaluated using QuBit 4 with the DNA High Sensitivity kit (Thermo Fisher Scientific) and fragment sizes were evaluated using Tapestation with the D1000 ScreenTape System kit (Agilent Technologies; Waldbronn, Germany). The genome was sequenced using the Illumina NextSeq 550 platform (Illumina). The reads were filtered to remove low quality sequences (<Q20) and those smaller than 35 bp using Fastp v0.19 (Chen et al. 2018). Then, the reads were assembled *de novo* using Megahit v1.2.9 program (Li et al. 2015) with the parameters in default mode and the assemblies were enhanced using CAP3 software (Huang and Madan 1999). Genome annotation was performed using RAST (Aziz et al. 2008). In addition, a search for genes related to bacteriocins and ribosomally synthesized and post-translationally modified peptides was performed using the BAGEL4 tool (van Heel et al. 2018), for genes related to secondary metabolites using antiSMASH 7.0 (Blin et al. 2023) and visual inspection. The tools of Center for Genomic Epidemiology (https://www.genomicepidemiology.org/services/) were used for: SCC*mec* typing (SCC*mec*Finder 1.2) and identification of acquired virulence genes for *S. aureus* (VirulenceFinder 2.0); and identification of acquired antibiotic resistance genes (ResFinder 4.1). Sequence types (ST) were determined using PubMLST (https://pubmlst.org/).

### Statistical analysis

Data were analyzed using GraphPad Prism software version 8.01 (Prism, GraphPad Software, San Diego, CA, USA) and the Fisher's exact test. Results were considered as statistically significant when *p* <0.05.

## Results

### AMS-screening and characteristics of the children colonized by AMS-producers

A total of 973 colonies were selected, 78.7% (700 from skin and 66 from nares) from the AD children (11 to 34 colonies per child; mean: 25.53; standard deviation: 7.52) and 21.3% (168 from skin and 39 from nares) from the non-AD group (14 to 20 colonies per child; mean: 17.08; standard deviation: 2.31). Of these colonies, 955 (98.1%) were identified as *Staphylococcus* and screened for AMS production: 306 *S. aureus*, 360 *S. epidermidis*, 137 *S. hominis*, 49 *S. saprophyticus*, 48 *S. capitis*, 21 *S. haemolyticus*, and 34 another CoNS (3 *S. arlattae*, 2 *S. caprae*, 19 *S. cohnii*, 2 *S. lugdunensis*, 1 *S. pasteuri*, and 7 *S. warneri*). All children were colonized by at least one species of *Staphylococcus* in each investigated site.

Fifty-seven (8.8%) CoNS isolates, mostly *S. epidermidis* (45.6%), colonizing the skin and nares of ten AD children and two non-AD children were able to reduce the growth of *S. aureus* ATCC29213. Some AD children were colonized by AMS-positive and AMS-negative colonies of the same species. There was no correlation with colonization by AMS-producing strains and gender, disease severity, or *S. aureus* absence (*P* > 0.05). In fact, a child with severe AD (child 4) was the one in which most AMS-positive colonies were detected, all of them *S. epidermidis*. This child was colonized by *S. aureus* on the lesional skin, where 10 AMS-positive colonies of *S. epidermidis* were identified (Table 1).

**Table 1.** Characteristics of the 12 children with and without AD in which AMS-producers were detected

Child	Age/gender	SCORAD	Isolation site	Species	colonies selected (n)	colonies AMS+ (n)
1	8/M	moderate	L	*S. aureus*	5	0
				*S. epidermidis*	10	0
			NL	*S. aureus*	5	0
				*S. epidermidis*	10	0
			N	*S. aureus*	1	**1**
				*S. epidermidis*	1	0
				*C. propinquum*	1	NA
4	6/F	severe	L	*S. aureus*	2	0
				*S. cohnii*	3	0
				*S. epidermidis*	10	**10**
			NL	*S. cohnii*	5	0
				*S. epidermidis*	10	**4**
			N	*S. aureus*	1	0
				*S. epidermidis*	1	0
6	7/F	moderate	L	*S. aureus*	5	0
			NL	*S. aureus*	5	0
			N	*S. aureus*	1	0
				*S. epidermidis*	1	0
				*S. lugdunensis*	1	**1**
9	5/F	moderate	L	*S. aureus*	5	0
			NL	*S. aureus*	5	0
				*S. epidermidis*	1	**1**
				*S. hominis*	2	0
			N	*S. aureus*	1	0
10	7/M	moderate	L	*S. aureus*	5	0
			NL	*S. aureus*	5	0
				*S. epidermidis*	5	**5**
				*S. haemolyticus*	1	0
				*S. hominis*	4	0
			N	*S. aureus*	1	0
				*S. epidermidis*	1	**1**
				*C. pseudodiphtheriticum*	1	NA
11	3/M	moderate	L	*S. aureus*	5	0
			NL	*S. epidermidis*	10	**9**
				*S. sciuri*	5	0
			N	*S. aureus*	1	0
				*S. epidermidis*	1	0
14	9/M	mild	L	*S. aureus*	5	0
				*S. epidermidis*	8	**1**
				*S. warneri*	2	0
			NL	*S. aureus*	3	0
				*S. capitis*	1	0
				*S. caprae*	2	0
				*S. epidermidis*	4	**1**
				*S. haemolyticus*	1	0
				*S. hominis*	3	0
			N	*S. aureus*	1	0
				*S. capitis*	1	0
19	6/F	moderate	L	*S. aureus*	5	0
				*S. epidermidis*	10	**1**
			NL	*S. aureus*	5	0
				*S. epidermidis*	7	0
				*S. hominis*	1	0
				*S. warneri*	2	0
			N	*S. aureus*	1	0
				*S. epidermidis*	1	0
				*C. pseudodiphtheriticum*	1	NA
24	9/M	mild	L	*S. aureus*	5	0
				*S. epidermidis*	10	0
			NL	*S. aureus*	2	0
				*S. epidermidis*	2	0
				*S. haemolyticus*	1	0
				*S. hominis*	2	0
				*S. warneri*	1	**1**
			N	*S. aureus*	1	0
				*S. warneri*	1	0
				*C. pseudodiphtheriticum*	1	NA
25	3/F	moderate	L	*S. aureus*	5	0
				*S. epidermidis*	8	0
				*S. hominis*	2	**2**
			NL	*S. aureus*	5	0
				*S. epidermidis*	5	0
				*S. hominis*	5	**4**
			N	*S. aureus*	1	0
				*S. epidermidis*	1	0
				*S. hominis*	1	0
SDA8	2/M	NA	H	*S. capitis*	2	0
				*S. epidermidis*	2	0
				*S. haemolyticus*	4	0
				*S. hominis*	6	0
				*S. saprophyticus*	1	0
			N	*S. epidermidis*	1	**1**
				*S. hominis*	1	0
				*S. haemolyticus*	1	0
				*S. saprophyticus*	1	0
				*C. pseudodiphtheriticum*	1	NA
SDA11	6/M	NA	H	*S. aureus*	5	0
				*S. epidermidis*	7	0
				*S. hominis*	3	0
			N	*S. aureus*	1	0
				*S. epidermidis*	1	0
				*S. lugdunensis*	1	**1**
				*C. pseudodiphtheriticum*	1	NA

AMS+—Positive for antimicrobial substance production; F—Female; H—Health skin; L—Lesional skin; NL—Non-lesional skin; M—Male; n—Number; N—Nares; NA—Not applicable; SCORAD—Scoring atopic dermatitis; SDA—Child without atopic dermatitis. The age is represented in years. Numbers in bold highlight the number of colonies positive for production of antimicrobial substance.

**Figure 1.**
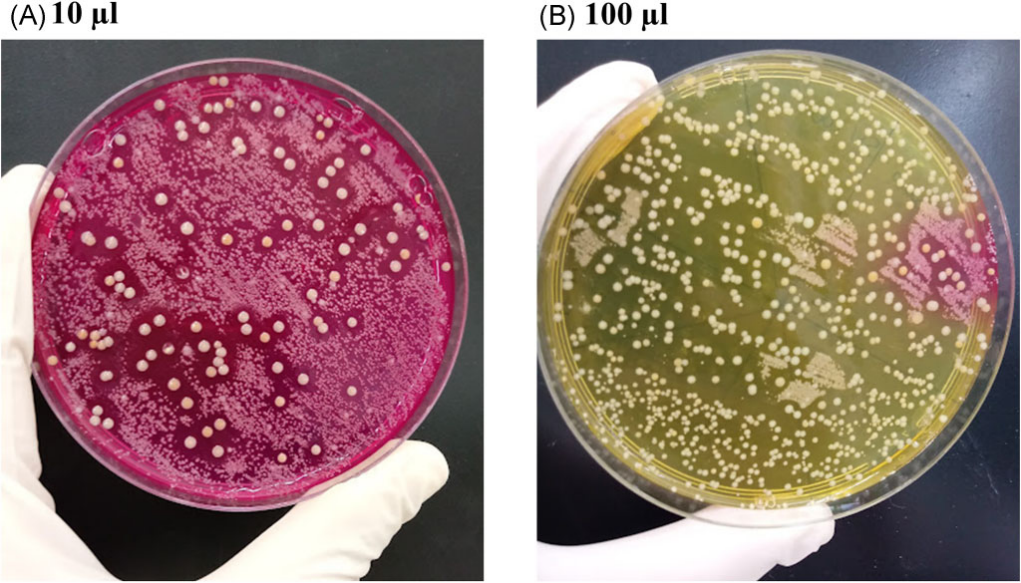
Mannitol salt agar plates in which 10 µL (A) and 100 µL (B) of the nasal swab suspensions of AD child 1 were inoculated. In (A), the larger colonies with inhibition zones around them were mannitol fermenters and they were subsequently identified as methicillin-resistant *Staphylococcus aureus*. The other colonies were identified as *Staphylococcus epidermidis* and *Corynebacterium propinquum*. On the 100 µL plate (B), as there is a larger amount of *Staphylococcus aureus* colonies, a drastic reduction in the amount of the smaller colonies is observed.

Only one child with moderate AD was colonized by an *S. aureus* isolate in the nares that inhibited the *S. epidermidis* ATCC12228 growth. This isolate has been previously characterized as MRSA/ST30/CC30 (Guimarães et al. 2022). Surprisingly, the mannitol-salt agar where this isolate was detected presented mannitol-fermenting colonies already showing inhibition zones (Fig. 1A, B). Of note is the fact that the number of mannitol non-fermenting colonies (identified as *Corynebacterium propinquum* and *S. epidermidis*) was reduced when a higher volume of the nares content was plated (Fig. 1B).

None of the 44 AMS-positive colonies had their action inhibited by NaOH, indicating that they were not metabolic acids. Therefore, we analyzed all the 44 colonies in the following experiments.

### Spectrum of action of the AMS

**Figure 2.**
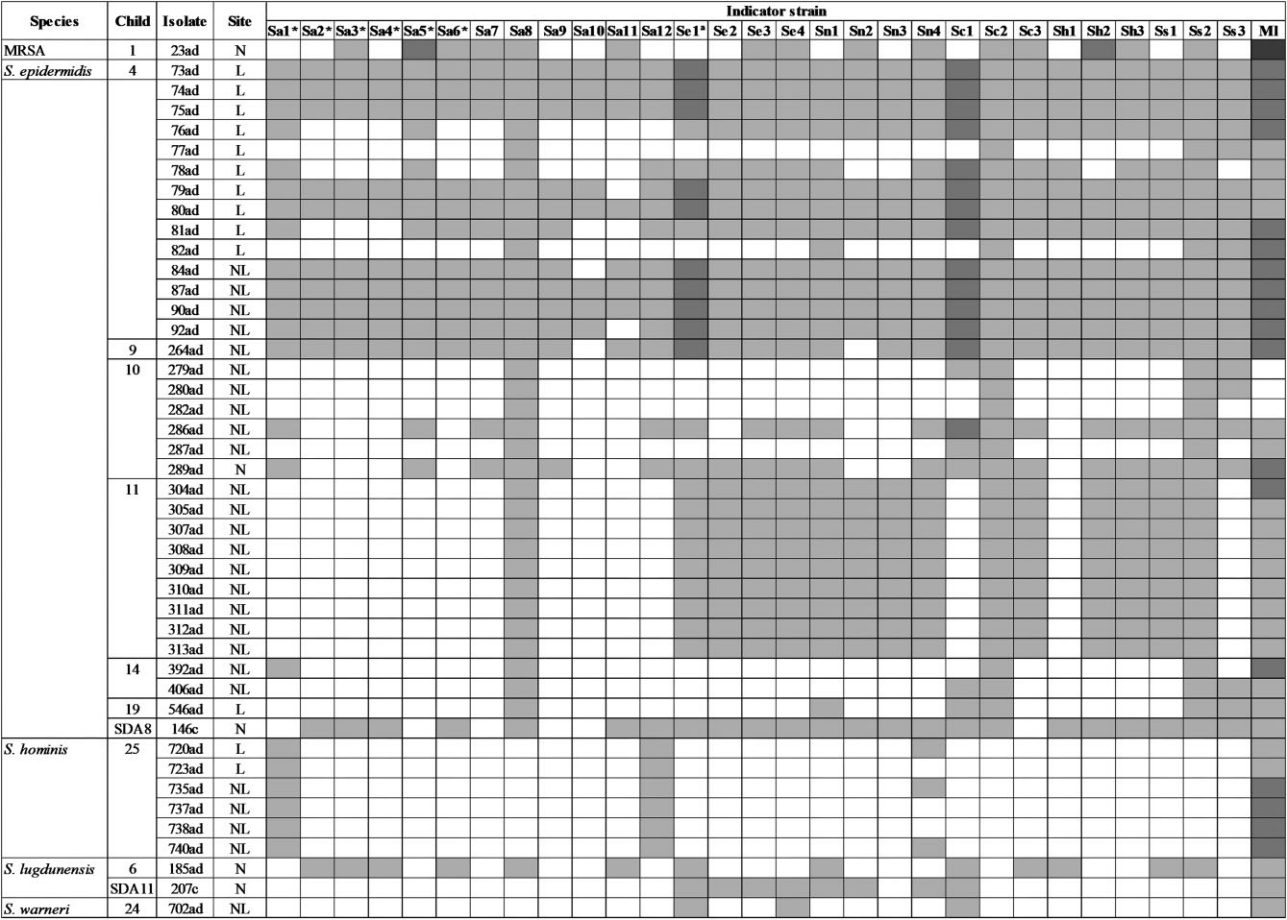
Heat map of the spectrum of action of the 44 AMS-producer isolates identified in this study. MRSA- Methicillin-resistant *Staphylococcus aureus*; ad—atopic dermatitis; c—control; N—Nares; L—Lesional skin; NL—Non-lesional skin; SDA—Child without atopic dermatitis; Sa*—Methicillin-resistant *Staphylococcus aureus*; Sa—Methicillin-sensitive *Staphylococcus aureus*; Se—*Staphylococcus epidermidis*; Sn—*Staphylococcus hominis*; Sc—*Staphylococcus capitis*; Sh—*Staphylococcus haemolyticus*; Ss—*Staphylococcus saprophyticus*; Ml—*Micrococcus luteus*. Distance border colony to border inhibition zone: 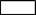 no inhibition 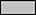 1–5 mm 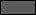 6–10 mm 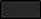 ≥ 10 mm.

Figure 2 shows the spectrum of action of each colony identified as an AMS-producer. Except for three colonies of the AD child 10, all AMS-producers inhibited the growth of *M. luteus*, with the MRSA isolate presenting the greater inhibition zone size. This MRSA isolate inhibited 15 of the 29 staphylococcal indicator strains (51.7%). The *S. epidermidis* colonies from AD children 4 and 9 were the ones presenting the broadest action. Indeed, six (42.8%) out of the 14 AMS-positive colonies detected in the AD child 4 inhibited all the indicator strains evaluated. It is also of note that the nine AMS-producing colonies of the AD child 11 inhibited 15 of the 29 *Staphylococcus* strains, with a more pronounced action against CoNS.

### Clonal evaluation of the AMS-producers

**Figure 3.**
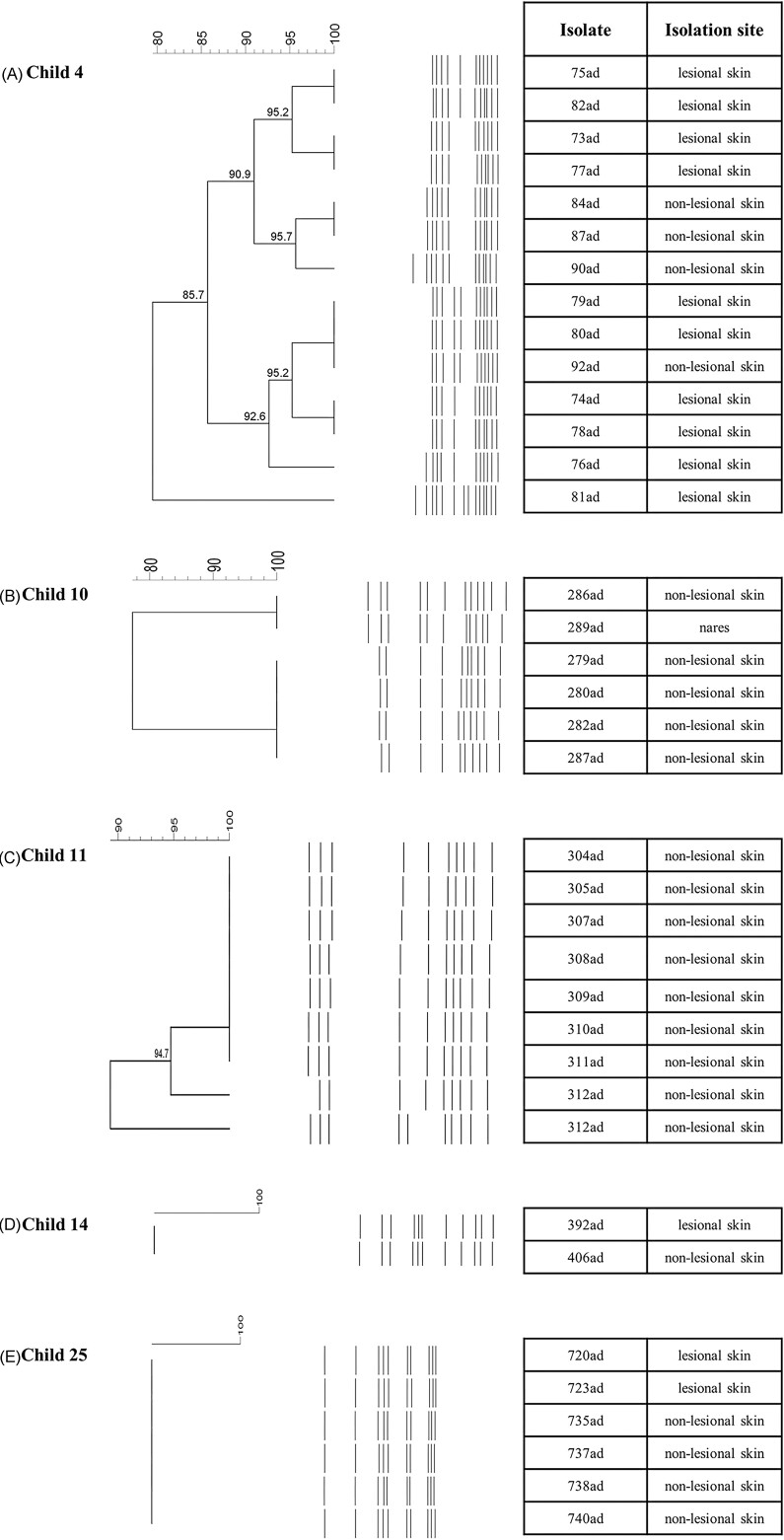
Dendrograms of AMS-positive colonies identified in five AD children. All isolates were *Staphylococcus epidermidis*, excepting the six from child 25 (all S. hominis).

Among the five AD children colonized by more than one colony producing AMS, three presented colonies of the same species with varied pulsotypes (AD children 4, 10, and 11; A, B and C), while the colonies of the two remaining children were of the same pulsotype (AD children 14 and 25; D and E) (Fig. 3). Interestingly, colonies of child 4 (A) presented the same pulsotypes (75ad and 82ad; 73ad and 77ad), but they inhibited the indicator strains differently (Fig. 3).

### Interaction between an AMS-positive *S. aureus* strain with members of the microbiota of the child in which it was isolated

**Figure 4.**
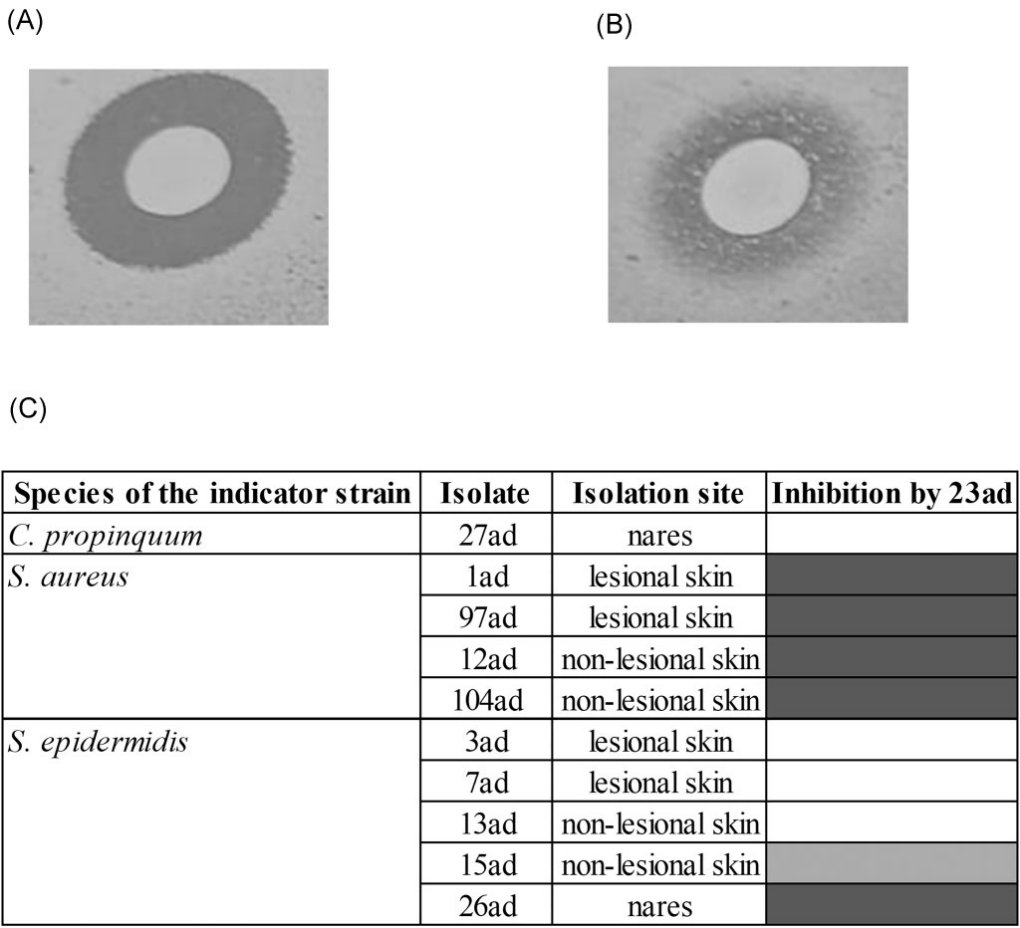
Spectrum of action of the AMS produced by MRSA 23ad against colonies of the AD child 1. (A) Inhibition of *S. aureus* 97ad; (B) Partial inhibition of *Staphylococcus epidermidis* 3ad; (C) Heat map of the spectrum of action of 23ad against 10 colonies. Distance border colony to border inhibition zone: 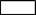 no inhibition 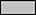 1–5 mm 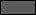 6–10 mm.

None of the 32 staphylococcal colonies selected from the child 1 with AD was able to inhibit the growth of MRSA 23ad isolated from nares of the same child. On the other hand, six isolates (*S. aureus* and *S. epidermidis*) of the ten (60%) evaluated of this child were sensitive (with clear inhibition zones) to the AMS produced by MRSA 23ad (Fig. 4A-C). It is noteworthy that this child was colonized by an *S. aureus* isolate of a distinct lineage in the lesional skin (*S. aureus* 1ad; MSSA ST333/CC15) (Guimarães et al. 2022) that was sensitive to the AMS of MRSA 23ad. Furthermore, *S. epidermidis* isolated from the nares of this child was sensitive to its action, while *Corynebacterium* was partially inhibited (without a clear inhibition zone).

### Detection of genes related to staphylococcal bacteriocins and auto-immunity

Thirty-two (72.7%) colonies were identified as producing proteinaceous AMS. Except for one isolate whose AMS was not sensitive to pronase (146c), all of them presented AMS sensitive to the three proteases. The isolates that did not present proteinaceous AMS were: 282ad, 287ad, 702ad, and all the nine colonies from the child 11 with AD. None of the proteinaceous AMS-producers presented any of the four bacteriocin genes evaluated. Except for one isolate (146c), all of them were resistant to the action of its own AMS.

### Genome sequencing of two AMS-producers

#### Genome of *S. aureus* 23ad

The MRSA 23ad isolate from the nares of a child with AD had a genome size of 2,817,018 bp and GC content of 32.8%. The genome was assembled into 51 contigs and presented 67 RNAs, including 59 tRNA and 8 rRNA encoding genes, corresponding to total transporter RNAs and ribosomal genes, respectively. When evaluating the gene sequence for the 16S rRNA in BLASTn, 100% identity with *Staphylococcus aureus* TCD2 (GCF_023716425.1) was observed.

After evaluation of the genome using the RAST tool, 2728 coding sequences were identified. Of these, 33% (n = 883) were categorized into subsystems, of which 844 were non-hypothetical. Most sequences categorized into subsystems were related to the synthesis or the metabolism of amino acids and derivatives (n = 233), carbohydrates (n = 176); and protein metabolism (n = 147). Among the sequences not categorized into subsystems were those related to methicillin resistance and one sequence related to colicin V.

**Figure 5.**
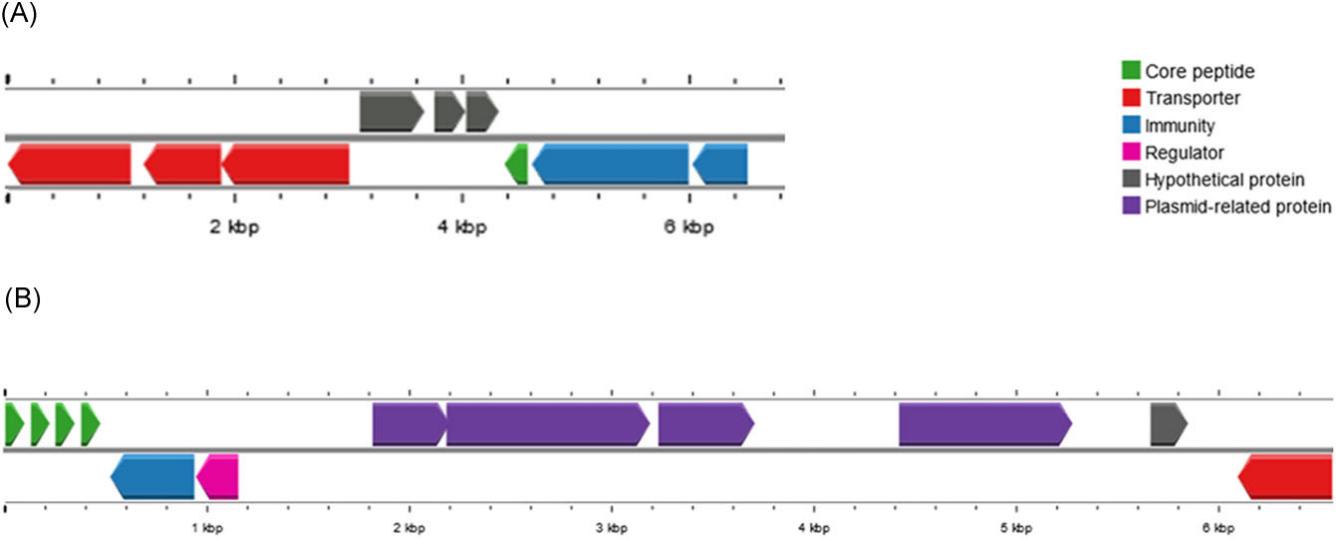
Genetic organization of bacteriocin genes clusters in *Staphylococcus epidermidis* 84 (SE84) and *Staphylococcus aureus* 23ad (SA23). (A) SE84–Cluster includes the gene encoding epidermicin NI01 variant (green arrow), transporters (red arrows), putative immunity proteins (blue arrow) and hypothetical protein (gray arrows). (B) SA23–Cluster of aureocin 4181 includes the four core peptides (green arrows), blue immunity protein (blue arrow), regulatory protein (pink arrow), partial transporter (red arrow). The unusual gene cluster organization reveals four plasmid-related genes (purple arrows) and a hypothetical protein (gray arrow).

When performing the genome analysis on the BAGEL4 and antiSMASH tools associated with the visual inspection only one complete bacteriocin gene cluster was identified (Fig. 5A). The gene cluster found encodes aureocin 4181, a variant of aureocin A70. The cluster is composed by four core peptides, one ABC transporter, one immunity gene and transcriptional regulator. The ABC transporter is on the edge of the contig 14#, and due to this, is incomplete. However, the partial amino acid sequence found have 100% identity to the protein aurT (data not shown). The product of the other genes found (*aurA, aurB, aurC, aurD1, aurI* and *aurR*) were 100% identical to genes found at the gene cluster of aureocin 4181.

In addition, using the antiSMASH 7.0.1 tool, four regions related to secondary metabolites were identified: two siderophores staphyloferrin A, terpene/T3PKS (type 3 polyketide synthases), staphylopine (opine-like-metallophore), and aureusimine (NRPS).

Genes related to methicillin (*mecA*) and trimethoprim (*dfrG*) resistance were detected in its genome, which carried the SCC*mec* type IVa. Virulence genes with 100% of identity were identified: *scn* (staphylococcal complement inhibitor), *splE* (serine protease splE), *hlgA* (gamma-hemolysin chain II precursor), *hlgB* (gamma-hemolysin component B precursor), *hlgC* (gamma-hemolysin component C), *sei* (enteroxin I), *seo* (enterotoxin O), and *seu* (enterotoxin U). Other virulence genes with more than 97% of identity and less than 100% detected were: *sak* (staphylokinase), *aur* (aureolysin), *seg* (enterotoxin G), *sem* (enterotoxin M), and *sen* (enterotoxin N). This isolate was from the ST30 and clonal complex 30.

#### Genome of *S. epidermidis* 84ad

Isolate 84ad from skin without lesion of a child with AD had a genome size of 2,496,819 bp and GC content of 31.9%. The genome was assembled into 45 contigs and presented 64 RNAs, including 59 tRNA and 5 rRNA encoding genes, corresponding to total transporter RNAs and ribosomal genes, respectively. When evaluating the gene sequence for the 16S rRNA in BLASTn, 100% identity was observed with *Staphylococcus epidermidis* SESURV_p4_1553 (GCF_011307715.1).

The genome was annotated using RAST, where 2409 coding sequences were detected. Among the coding sequences, 34% (n = 806) were categorized into subsystems, of which 771 were non-hypothetical. Most of the sequences categorized into subsystems were related to synthesis or the metabolism of amino acids and derivatives (n = 204), carbohydrates (n = 163); and protein metabolism (n = 132). A sequence related to colicin V was also observed.

Genome analysis on the BAGEL4 and AntiSMASH platforms resulted in the detection of a subregion of 6 kb with a similar genetic organization (98% similarity) to epidermicin NI01, which is a leaderless bacteriocin produced by *Staphylococcus epidermidis* strain 224 (Sandiford and Upton 2012). The homolog genes identified in *S. epidermidis* 84ad were: *ydbS*-like and *ydbT*-like that encode proteins responsible by self-protection, the structural *edcA* gene of epidermicin NI01 related with the lacticin Z (Iwatani et al. 2007), another leaderless bacteriocin, three hypothetical protein genes, RND efflux transporter and two ABC transporters (Fig. 5). The core peptide present is a one amino acid variant of epidermicin NI01.

In addition, using the antiSMASH 7.0.1 platform, three secondary metabolite clusters regions were detected: T3PKS, siderophore, and a non-ribosomally synthesized peptide. Even though this isolate did not present the *mecA* gene, genes related to antimicrobial resistance were identified to the classes: aminoglycoside (*aadD, aph*(3′)-III, *ant(6)-Ia*), streptogramin b (*msr(A)*), beta-lactam (*blaZ*), fosfomycin (*fosB*), lincosamide (*lnu(A)*), macrolide (*mph(C), mrs(A)*), and tetracycline (*tetK*). This isolate belonged to ST558.

## Discussion

Although studies point to the ability of CoNS to inhibit *S. aureus* colonizing individuals with (Nakatsuji et al. 2017, Nakatsuji et al. 2021a) and without atopic dermatitis (Janek et al. 2016, Lynch et al. 2019, O'Sullivan et al. 2019b, Liu et al. 2020), the interaction of CoNS with other members of the microbiota is little discussed in this disease. Also, the action of *S. aureus* against CoNS isolates from AD had not yet been addressed. In the present study, we detected AMS-producing CoNS colonizing 10 children with AD (33.3% of children with AD) and two without the disease (17.7% of children without AD), mostly *S. epidermidis* (45.6%). In addition, to the best of our knowledge, for the first time an AMS-producing MRSA strain was identified colonizing the nares of a child with AD. Therefore, the study provides an interesting contribution to the interrelationship and competition of different species of staphylococci in the skin and nostrils of children with AD and without the disease.

*Staphylococcus* spp. have been shown to produce bacteriocins with action against other staphylococcal and Gram-positive species (Bastos et al. 2020). Nisin J, for example, is a bacteriocin detected in *S. capitis* isolated from skin of a healthy human that inhibits strains of *S. aureus* (MSSA and MRSA), *S. epidermidis, S. simulans*, and Gram-positive bacteria from other genera (O'Sullivan et al. 2019a). The present study is possibly the first to detect isolates presenting antimicrobial activity against different bacterial species from the skin of children with AD. In addition, some of the AMS-positive CoNS isolates showed larger inhibition zones when tested against CoNS. It is also worth noting that AMS-producer isolates were coexisting at the same site as AMS-negative colonies that were completely or partially inhibited by them. These results seem to indicate that the presence of AMS-producing colonies does not completely eradicate other members of the microbiota, but it possibly controls the abundance of certain strains and favors their permanence by promoting competition. Therefore, this finding adds to the questioning about the increase in the population of *S. epidermidis* together with *S. aureus* when the disease is exacerbated in some cases of atopic dermatitis (Kong et al. 2012, Byrd et al. 2017), not only to control *S. aureus*, but also in relation to the inhibition of other members of the microbiota, possibly contributing indirectly to dysbiosis. Therefore, maintenance microbial balance appears crucial in children with AD, in whom even a species often considered as beneficial is reported causing skin damage (Cau et al. 2020).

It is worth noting that other studies have reported *S. aureus* isolates producing AMS (Daly et al. 2010, Joo et al. 2011, Janek et al. 2016, Marques-Bastos et al. 2020, Kassem et al. 2021), but little is known about their presence in humans and their interaction with members of the microbiota (Daly et al. 2010, Joo et al. 2011, Janek et al. 2016, Kassem et al. 2021). Janek and colleagues (2016) et al. (2016) isolated *S. aureus* strains producing AMS from the nares of healthy adults but did not observe action against *S. aureus* and *S. epidermidis* strains. We believe that this is the first report of the detection of an MRSA isolate colonizing the nares of an AD child producing AMS, and one of the few studies demonstrating its interaction with members of the human skin microbiota. Since *S. aureus* can be transferred from a site to another in the same individual (Guimarães et al. 2022), nasal colonization by AMS-positive MRSA may be a risk factor for its spread to the skin and may negatively impact the course and therapy of AD, since MRSA strains represent a major therapeutic problem and are often associated with more severe cases of AD.

Although only one of the *S. epidermidis* colonies evaluated from the skin of child 1 with AD was completely sensitive to the action of the AMS-positive *S. aureus* (MRSA ST30/CC30), all the *S. aureus* colonies evaluated from the skin of this child were sensitive (one of the isolates was characterized as Methicillin-sensitive *S. aureus* [MSSA] ST333/CC15). It is noteworthy that, in addition to producing a proteinaceous AMS (possibly a bacteriocin), this is a MRSA strain, and its transfer to the skin can lead to difficulties in treatment in case of an infection. This MRSA was also able to inhibit staphylococcal species isolated from other children, such as *S. hominis*, which were not detected in this child, but could be present in low abundance, which could further contribute to the dysbiosis. Therefore, we hypothesize that another possible explanation for abundance of *S. aureus* during crisis, in some AD patients, could be due to production of AMS inhibiting other members of the microbiota.

The presence of a mixed population colonizing humans containing both AMS-producing and non-producing strains was observed by other authors (Nakatsuji et al. 2017, Lee et al. 2019, O'Sullivan et al. 2019b). O'Sullivan and colleagues (2019b) reported that the same individual carried more than one strain of the same species and the same genotype by PFGE producing AMS at different skin sites. As in our study, the authors also observed some strains of the same species isolated from different sites of the same individual presenting different pulsotypes and varied sensitivities to substances produced by other AMS-positive isolates (O'Sullivan et al. 2019b). However, in our study, PFGE was unable to discriminate AMS-producing strains, in which some colonies with the same genotypic profile showed a distinct spectrum of action. Therefore, AMS production by *Staphylococcus* spp. colonizing the skin and nares of human beings appears to be strain-dependent, which reinforces the need for evaluation of multiple colonies regardless of their genotypic profile.

It should also be noted that detection of a novel AMS is not uncommon when evaluating AMS production by *Staphylococcus* isolated from humans (Janek et al. 2016, Zipperer et al. 2016, Nakatsuji et al. 2017, Lynch et al. 2019, O'Sullivan et al. 2019b). Nakatsuji and colleagues (2017) sequenced the genome of some AMS-producing CoNS isolates and detected genes for previously described bacteriocins using the RAST tool, such as Pep5 and epidermin, and described two new lantibiotics produced by *S. hominis*, the lantibiotic Sh-α and Sh-β. Consequently, we selected two protein AMS-producing isolates detected in our study and sequenced their genome (*S. aureus* 23ad and *S. epidermidis* 84ad) to search for bacteriocin-related genes and other AMS previously described.

Regarding the strain MRSA 23ad genome, the gene cluster probably related to it inhibitory activity is the one encoding the bacteriocin aureocin 4181 (Marques-Bastos et al. 2020). Although the genes and partial *aur*T gene found were 100% identical to aureocin 4181, the genetic organization is different (Fig. 5). The partial *aurT* gene is not next to *aur*A gene and it is separated from the bacteriocinogenic cluster by five genes, four plasmid-related genes and one with unknow function. Further studies must be done in order to understand if this organization affects the production of the peptides.

Similarly, in the *S. epidermidis* 84ad genome sequenced by our group, the presence of the *edcA* gene of epidermicin NI01 (related to peptide lacticin Z) had already been described (Sandiford and Upton 2012), which also exhibited potent antimicrobial activity against Gram-positive bacteria, including MRSA strains. It is important to highlight that this is a new variant of epidermicin NI01, which presents a substitution of the amino acid in position 16, from a Gln to a Lys.

Although the presence of genes related to one or more bacteriocins is reported in the genome of *Staphylococcus* spp., it should be noted that gene clusters are not always functional or expressed (Bastos et al. 2020). Thus, further analysis is needed to confirm whether these isolates produce the bacteriocins already described (or a variant of these bacteriocins) or whether the activity observed in this study is due to the presence of another molecule (s).

Similar to what was found by Cohen and collaborators (2018) in *Escherichia coli* isolates from human stool, a gene related to the colicin V was also detected in our isolates. The authors, through knockout experiments, showed that the *cvaC* gene was responsible for encoding the colicin V bacteriocin (Cohen et al. 2018). However, Wang and coworkers (2023) after experiments involving mutagenesis in *S. aureus* RMSA24 isolated from a raw milk sample demonstrated that colicin V gene is associated with resistance of the pathogen to desiccation stress (Wang et al. 2023).

Regarding limitations in detecting AMS-producing colonies, the low number of children investigated in our study and the low number of selected colonies per site (compared to the number evaluated by Nakatsuji et al. 2017) may have limited the broad comparison of frequency of inhibitory strains colonizing each group and each site.

In conclusion, AMS production by CoNS was able to inhibit *S. aureus* strains and other species in the skin microbiota. Of note is the detection of a MRSA colonizing a child with AD with antimicrobial action against different species of staphylococci. This fact reinforces the virulent role of *S. aureus*, which is abundant in these patients contributing to dysbiosis and the severity of the disease.

## Supplementary Material

fiae070_Supplemental_File
