# Correction to: Methicillin-resistant *Staphylococcus aureus* and coagulase-negative *Staphylococcus* produce antimicrobial substances against members of the skin microbiota in children with atopic dermatitis

**DOI:** 10.1093/femsec/fiae107

**Published:** 2024-08-13

## Abstract

Globally abundant open ocean and coastal ocean *Synechococcus* strains preferentially utilize phosphoanhydrides over phosphoesters.

This is a correction to: Lorrayne Cardoso Guimarães, Gizele Duarte Garcia, Fernanda Sampaio Cavalcante, Graciela Maria Dias, Felipe Miceli de Farias, Simone Saintive, Eliane de Dios Abad, Dennis de Carvalho Ferreira, Kátia Regina Netto dos Santos, Methicillin-resistant *Staphylococcus aureus* and coagulase-negative *Staphylococcus* produce antimicrobial substances against members of the skin microbiota in children with atopic dermatitis, FEMS Microbiology Ecology, Volume 100, Issue 6, June 2024, https://doi.org/10.1093/femsec/fiae070

In the originally published version of this manuscript, there was an error in the following sentence in the section entitled “Clonal evaluation of AMS-producers”, whereby “<” should have been given as “≥”:

Isolates presenting a similarity coefficient < 80% were assigned as the same genotype (Van Belkum et al. 2009).

The sentence has been corrected to read as follows:

Isolates presenting a similarity coefficient ≥ 80% were assigned as the same genotype (Van Belkum et al. 2009).

