# Supplementary material for: Methicillin-resistant Staphylococcus aureus and coagulase-negative Staphylococcus produce antimicrobial substances against members of the skin microbiota in children with atopic dermatitis
Source: FEMS Microbiol Ecol. 2024 May 28;100(6):fiae070. doi: 10.1093/femsec/fiae070 (PMC11141783; doi:10.1093/femsec/fiae070)
Supplement: fiae070_Supplemental_File [file fiae070_supplemental_file.doc]

**Supplementary Table 1- Description of the indicator strains used to evaluate the spectrum of action of AMS produced by *Staphylococcus* isolated from the skin and nares of children with and without AD**

| **Species** | **Indicator strain** | **Identification** | **Methicillin susceptibility** | **Isolation site** | **Reference** |
| --- | --- | --- | --- | --- | --- |
| *M. luteus* | Ml | ATCC4698 | NA | NA | ATCC |
| *S. aureus* | Sa1 | ATCC33591 | MRSA | NA | ATCC |
|  | Sa2 | 29ad | MRSA | L (child 2) | Guimarães et al., 2022 |
|  | Sa3 | 118ad | MSSA | L (child 4) | Guimarães et al., 2022 |
|  | Sa4 | 223ad | MRSA | L (child 8) | Guimarães et al., 2022 |
|  | Sa5 | 253ad | MSSA | L (child 9) | Guimarães et al., 2022 |
|  | Sa6 | 268ad | MSSA | L (child 10) | Guimarães et al., 2022 |
|  | Sa7 | 317ad | MRSA | L (child 12) | Guimarães et al., 2022 |
|  | Sa8 | 383ad | MSSA | L (child 14) | Guimarães et al., 2022 |
|  | Sa9 | 538ad | MSSA | L (child 19) | Guimarães et al., 2022 |
|  | Sa10 | 684ad | MRSA | L (child 24) | Guimarães et al., 2022 |
|  | Sa11 | 711ad | MSSA | L (child 25) | Guimarães et al., 2022 |
|  | Sa12 | 788ad | MSSA | L (child 28) | Guimarães et al., 2022 |
| *S. epidermidis* | Se1 | ATCC12228 | MS-CoNS | NA | ATCC |
|  | Se2 | 3ad | MR-CoNS | L (child 1) | Guimarães et al., 2022 |
|  | Se3 | 85ad | ND | NL (child 4) | This study |
|  | Se4 | 716ad | ND | L (child 25) | Guimarães et al., 2022 |
| *S. hominis* | Sn1 | ATCC27844 | MS-CoNS | NA | ATCC |
|  | Sn2 | 234ad | MR-CoNS | NL (child 8) | Guimarães et al., 2022 |
|  | Sn3 | 404ad | ND | NL (child 14) | Guimarães et al., 2022 |
|  | Sn4 | 561ad | MR-CoNS | NL (child 19) | Guimarães et al., 2022 |
| *S. capitis* | Sc1 | ATCC27840 | MS-CoNS | NA | ATCC |
|  | Sc2 | 408ad | ND | NL (child 14) | Guimarães et al., 2022 |
|  | Sc3 | 606ad | ND | NL (child 21) | Guimarães et al., 2022 |
| *S. haemolyticus* | Sh1 | ATCC29970 | MS-CoNS | NA | ATCC |
|  | Sh2 | 61ad | ND | NL (child 3) | Guimarães et al., 2022 |
|  | Sh3 | 399ad | MR-CoNS | NL (child 14) | Guimarães et al., 2022 |
| *S. saprophyticus* | Ss1 | ATCC15305 | MS-CoNS | NA | ATCC |
|  | Ss2 | 228ad | ND | NL (child 8) | Guimarães et al., 2022 |
|  | Ss3 | 521ad | MR-CoNS | NL (child 18) | Guimarães et al., 2022 |

Ml- *Micrococcus luteus*; MRSA- Methicillin-resistant *Staphylococcus aureus*; MR-CoNS- Methicillin-resistant coagulase-negative *Staphylococcus*; MSSA- Methicillin-sensitive *Staphylococcus aureus*; MS-CoNS- Methicillin-sensitive coagulase-negative *Staphylococcus*; NA- Not applicable; ND- Not determined; L- Lesional skin; NL- Non-lesional skin. N- Nares; Sa- *Staphylococcus aureus*; Se- *Staphylococcus epidermidis*; Sn- *Staphylococcus hominis*; Sc- *Staphylococcus capitis*; Sh- *Staphylococcus haemolyticus*; Ss- *Staphylococcus saprophyticus*.

**Supplementary table 2- Description of the bacterial isolates from AD child 1 evaluated for their susceptibility to the AMS produced by MRSA 23ad**

| **Species** | **Isolate(s)** | **Isolation site** | **Reference** |
| --- | --- | --- | --- |
| *Corynebacterium propinquum* | 27ad | nares | This study |
| *S. aureus* | 1ad^a^ | lesional skin | Guimarães et al., 2022 |
|  | 97ad | lesional skin | This study |
|  | 12ad | non-lesional skin | This study |
|  | 104ad | non-lesional skin | This study |
| *S. epidermidis* | 3ad | lesional skin | This study |
|  | 7ad | lesional skin | This study |
|  | 13ad | non-lesional skin | This study |
|  | 15ad | non-lesional skin | This study |
|  | 26ad | nares | This study |

^a^ Methicillin-sensitive *Staphylococcus aureus* presenting sequence type 333 and from clonal complex 15.

**Supplementary table 3 - Description of the 14 bacterial isolates selected for the auto-immunity assay**

| **Child** | **Isolate** | **Isolation site** | **Reference** |
| --- | --- | --- | --- |
| 1 | 23ad | nares | This study |
| 4 | 73ad | lesional skin | This study |
| 4 | 84ad | non-lesional skin | This study |
| 6 | 185ad | nares | This study |
| 9 | 264ad | non-lesional skin | This study |
| 10 | 280ad | non-lesional skin | This study |
| 10 | 289ad | nares | This study |
| 14 | 392ad | lesional skin | This study |
| 14 | 406ad | non-lesional skin | This study |
| 19 | 546ad | lesional skin | This study |
| 25 | 720ad | lesional skin | This study |
| 25 | 735ad | non-lesional skin | This study |
| SDA8 | 146c | nares | This study |
| SDA11 | 207c | nares | This study |

SDA- Child without atopic dermatitis.
